# Combination Therapy with the Histone Deacetylase Inhibitor LBH589 and Radiation Is an Effective Regimen for Prostate Cancer Cells

**DOI:** 10.1371/journal.pone.0074253

**Published:** 2013-08-26

**Authors:** Weiwei Xiao, Peter H. Graham, Jingli Hao, Lei Chang, Jie Ni, Carl A. Power, Qihan Dong, John H. Kearsley, Yong Li

**Affiliations:** 1 Cancer Care Centre and Prostate Cancer Institute, St George Hospital, Kogarah, New South Wales, Australia; 2 Faculty of Medicine, University of New South Wales, Kensington, New South Wales, Australia; 3 Department of Radiation Oncology, Cancer Centre, Sun Yat-sen University, Guangzhou, Guangdong, China; 4 State Key Laboratory of Oncology in Southern China, Guangzhou, Guangdong, China; 5 Biological Resources Imaging Laboratory, University of New South Wales, New South Wales, Australia; 6 School of Science and Health Science, University of Western Sydney, New South Wales, Australia; 7 Department of Endocrinology, Royal Prince Alfred Hospital and Bosch Institute, The University of Sydney, New South Wales, Australia; University of Colorado, United States of America

## Abstract

Radiation therapy (RT) continues to be one of the most popular treatment options for localized prostate cancer (CaP). The purpose of the study was to investigate the *in vitro* effect of LBH589 alone and in combination with RT on the growth and survival of CaP cell lines and the possible mechanisms of radiosensitization of this combination therapy. The effect of LBH589 alone or in combination with RT on two CaP cell lines (PC-3 and LNCaP) and a normal prostatic epithelial cell line (RWPE-1) was studied by MTT and clonogenic assays, cell cycle analysis, western blotting of apoptosis-related and cell check point proteins, and DNA double strand break (DSB) repair markers. The immunofluorescence staining was used to further confirm DSB expression in treated CaP cells. Our results indicate that LBH589 inhibited proliferation in both CaP and normal prostatic epithelial cells in a time-and-dose-dependent manner; low-dose of LBH589 (IC_20_) combined with RT greatly improved efficiency of cell killing in CaP cells; compared to RT alone, the combination treatment with LBH589 and RT induced more apoptosis and led to a steady increase of sub-G1 population and abolishment of RT-induced G2/M arrest, increased and persistent DSB, less activation of non-homologous end joining (NHEJ)/homologous recombination (HR) repair pathways and a panel of cell cycle related proteins. These results suggest that LBH589 is a potential agent to increase radiosensitivity of human CaP cells. LBH589 used either alone, or in combination with RT is an attractive strategy for treating human CaP.

## Introduction

Current treatment options for localized CaP are radiation therapy (RT), surgery and endocrine therapy. Although aggressive radiation does improve biochemical control, greater rectal and urinary toxicities also occurred [1]. Local failure after RT remains 20%–35% in intermediate- and high-risk CaP patients [2], leading to increased metastasis and lower survival. Thus, investigation of a novel combination approach with a selective radio-sensitizer with RT to enhance CaP radiosensitivity is urgently needed.

Histone deacetylase inhibitors (HDACi) are an emerging group of agents which targets histone deacetylase (HDAC) and promising radiosensitizers currently under investigation. Radiosensitization by HDACi, such as valproic acid [3] has been demonstrated in preclinical studies. HDACi is a potent inducer or regulator of cellular behaviours such as apoptosis, cell cycle and DNA repair processes. It is believed to exert its effects mainly by modifying histone and chromatin structures, thus modulate gene transcription [4]. Moreover, these acetylases and deacetylases can also modulate cell functions independent of gene expression by acting on non-histone proteins such as p21 [5], p53 [6], Ku70 [6]. Through acting on a series of histone and non-histone proteins, HDACi is capable of mediating apoptosis, cell cycle, and DNA repair processes in a well orchestrated manner.

LBH589 is a hydroxamic acid derivative and a novel pan-HDACi [7]. Qian et al. reported that LBH589 alone reduced angiogenesis and tumor growth in a PC-3 xenograft animal model [8]. A phase I study has been conducted by treating castration-resistant prostate cancer (CRPC) patients using oral LBH589 with or without docetaxel, demonstrating promising results for future clinical application [9]. These results support the hypothesis that LBH589 may be useful in combination with RT for treating localized CaP.

In this study, we hypothesized that LBH589 could kill CaP cells and treatment of CaP cells with LBH589 before RT would increase the sensitivity of CaP cells to RT.

## Materials and Methods

### Chemicals and antibodies

LBH589 (panobinostat) was purchased from Selleck Chemicals (Selleck Chemicals South Loop West, Houston, TX, USA). Other chemicals used were purchased from Sigma-Aldrich (Sigma-Aldrich, Pty Ltd, Castle Hills, NSW, Australia), unless specified otherwise. Primary and secondary antibodies used in this study are listed in Table **1**.

**Table 1:** Antibodies used for western blotting and immunofluorescence staining.

**Antibody**	**Source**	**Type**	**Dilution**	**Incubation time**	**Temperature**	**Application**
Rabbit anti-human Caspase-3 (Pro)	Epitomics	MAb	1:1000	o/n	4^°^C	WB
Rabbit anti-human Caspase-3 (Active)	Epitomics	MAb	1:1000	o/n	4^°^C	WB
Rabbit anti-human Histone H3 (acetyl K9)	Abcam	PAb	1:500	o/n	4^°^C	WB
Rabbit anti-human Histone H4 (acetyl K8)	Abcam	PAb	1:500	o/n	4^°^C	WB
Rabbit anti-human p21	Abcam	PAb	1:1000	o/n	4^°^C	WB
Rabbit anti-human p-p53	Abcam	PAb	1:1000	o/n	4^°^C	WB
Mouse anti-human p53	Abcam	MAb	1:1000	o/n	4^°^C	WB
Rabbit anti-human phospho-CDK1 (Tyr15)	Cell Signaling Technology	PAb	1:1000	o/n	4^°^C	WB
Rabbit anti-human Cdk1 (cdc2)	Abcam	PAb	1:10000	o/n	4^°^C	WB
Rabbit anti-human p-Chk-1	Abcam	PAb	1:1000	o/n	4^°^C	WB
Rabbit anti-human Chk-1	Abcam	PAb	1:1000	o/n	4^°^C	WB
Rabbit anti-human p-Chk-2	Abcam	PAb	1:500	o/n	4^°^C	WB
Rabbit anti-human Chk-2	Abcam	PAb	1:500	o/n	4^°^C	WB
Rabbit anti-human phospho-Rb (Ser795)	Cell Signaling Technology	PAb	1:1000	o/n	4^°^C	WB
Rabbit anti-human phospho-Rb (Ser807/811)	Cell Signaling Technology	PAb	1:1000	o/n	4^°^C	WB
Mouse anti-human Rb	Abcam	MAb	1:1000	o/n	4^°^C	WB
Mouse anti-human γH2AX	Abcam	MAb	1:500 (IF) 1:1000 (WB)	o/n	4^°^C	IF WB
Rabbit anti-human Ku70	Epitomics	PAb	1:1000	o/n	4^°^C	WB
Rabbit anti-human Ku80	Epitomics	PAb	1:1000	o/n	4^°^C	WB
Mouse anti-human BRCA1	Abcam	MAb	1:200 (IF) 1:200 (WB)	o/n	4^°^C	WB, IF
Rabbit anti-human BRCA2	Abcam	PAb	1:200 (IF) 1:1000 (WB)	o/n	4^°^C	WB, IF
Mouse anti-human RAD51	Abcam	PAb	1: 100 (IF) 1:1000 (WB)	o/n	4^°^C	WB, IF
Mouse anti-human GAPDH	Millipore	MAb	1:500	o/n	4^°^C	WB
Mouse anti-human IgG1-negative control	Dako	IgG1	1:1000	o/n	4^°^C	IF
Goat anti-rabbit IgG-HRP	Santa Cruz Biotechnology	IgG	1:5000	45 min	room temperature	WB
Goat anti-mouse IgG-HRP	Santa Cruz Biotechnology	IgG	1:5000	45 min	room temperature	WB
Goat anti-mouse Alexa Fluor® 488 Dye Conjugate	Invitrogen	IgG	1:1000	45 min	room temperature	IF

Notes: MAb: monoclonal antibody; o/n: overnight; PAb: polyclonal antibody; WB: western blotting; IF: immunofluorescence; HRP: horseradish peroxidas

### Cell culture

The androgen-non-responsive PC-3 and androgen-responsive LNCaP CaP cell lines, and the normal human prostate RWPE-1 cell line were obtained from American Type Culture Collection (ATCC) (Rockville, MD, USA). PC-3 and LNCaP cells were cultured in RPMI-1640 supplemented with 10% (vol/vol) heated-inactivated fetal bovine serum (FBS), 50 U/mL of penicillin, and 50 µg/mL of streptomycin while RWPE-1 cells were cultured in K-SFM medium supplemented by 0.2 ng/mL recombinant epidermal growth factor (rEGF) and 25 µg/mL bovine pituitary extract without FBS. All cell lines were maintained in a humidified incubator at 37°C and 5% CO_2_.

### MTT assay

Cell proliferation was evaluated in CaP and normal prostate cell lines after LBH589 treatment using MTT assay following a published method [10]. Briefly, 2000 cells were seeded in 96-well plates incubated in culture media for 24 h. Cells were then treated with a range of concentrations of LBH589 (0 ~ 20 µmol/L) or the same volume of DMSO control in fresh media for another 24, 48 and 72 h, respectively. The absorbance (OD) was read at 560 nm on a BIO-TEC micro-plate reader (BIO-RAD, Hercules, CA, USA). Each experiment was repeated at least three times. Results are represented as the OD ratio of the treated and control cells. IC_20_ (20% inhibitory concentration) of LBH589 at 24 h was calculated and chosen for the following experiments.

### Colony forming assay

PC-3, LNCaP and RWPE-1 cells were used for colony forming assays as described previously with minor modifications [10]. Briefly, 1.5~2×10^6^ PC-3, LNCaP and RWPE-1 cells were treated in 6 cm dish with LBH589 at the respective IC_20_ concentrations or same volume of DMSO (control) for 24 h and then 200~2×10^5^ cells were seeded in 6 cm dishes. After 8 h recovery, the LBH589-treated and DMSO-treated control cells were exposed to a single dose irradiation (0-8 Gy) at room temperature using a linear accelerator (Elekta, Stockholm, Sweden) at a dose rate of 2.7 Gy/min with 6 MV photons (Cancer Care Centre, St George Hospital, Sydney, Australia). After RT, all cells were immediately washed with drug-free medium. After 14 days incubation, cells were stained with crystal violet. The colonies, defined as groups of >50 cells, were scored manually with the aid of an Olympus INT-2 inverted microscope (Tokyo, Japan). Data from RT alone or combination treated (LBH589 and RT) cells were normalized against the un-irradiated cells (scored as 100% colony forming ability).

### Flow cytometric analysis for cell cycle distribution

0.5~2×10^6^ PC-3 and LNCaP CaP cells were plated in 10 cm dish for 24 h, then treated with LBH589 at the respective IC_20_ concentrations or DMSO (control) for another 24 h before subjected to 2 Gy RT. At specific time points (pre-RT, 2, 6, 12, 24, 48 and 72 h after 2 Gy), trypsinized adherent and floating cells were pooled and fixed in cold 70% (v/v) ethanol at 4°C. Histograms of DNA content were analysed using FlowJo software (V.7.6.1, Tree Star, Inc., Oregon, USA) to determine cell cycle distribution (subs G1, G1, S, and G2/M).

### Immunofluorescent staining for γH2AX and HR repair pathway proteins

PC-3 and LNCaP CaP cells were treated with the same protocol as described in cell cycle analysis (see above). After receiving 2 Gy RT, LBH589-treated and DSMO-treated cells were subjected to immunofluorescence staining at specific time points (pre-RT, 24, and 72 h after 2 Gy). The cell preparation for immunofluorescence staining was as previously described with modifications [11,12]. The stained cells were examined using an FV300/FV500 Olympus laser scanning confocal microscope (Olympus, Tokyo, Japan) and images were captured. For each treatment condition, γH2AX, BRCA1, BRCA2 and RAD51 foci were determined in at least 50 cells. The counting of γH2AX, BRCA1, BRCA2, and RAD51 nuclear foci in these cells was performed by two independent observers (WWX and JLH).

### Western blotting

PC-3 and LNCaP CaP cells were treated with the same protocol as described in cell cycle analysis (see above). LBH589-treated and control cells were subjected to 2 Gy RT. Both floating and adherent cells were collected at specific time points (pre-RT, 2, 6, 12, 24, 48 and 72 h after 2 Gy) for western blotting as described previously [13]. Membranes were incubated with different primary antibodies and horseradish peroxidise (HRP)-labelled secondary antibodies at specific conditions (Table **1**). ImageQuant LAS4000 system (GE Health care, USA) was used for image recording.

### Statistical analysis

All experiments were performed at least three times (n=3). Results are presented as mean± standard deviation (SD). For irradiation experiments, survival fractions were calculated as followed: SF = [mean plating efficiency of radiation (±LBH589) treated cells divided by mean plating efficiency of control (±LBH589)] % Survival fractions of combination-treated cells were corrected for the cytotoxicity of LBH589. RT survival curves were fitted according to the linear-quadratic model using GraphPad Prism 4.0 software (GraphPad, San Diego CA): *Survival = e^-(αD+βD^2^^)*

The following parameters were calculated: α value, β value, α/β value, ID90, ID_50_, and ID10 (RT dose producing a surviving fraction of 10%, 50%, and 90%, respectively); and SF2 (surviving fraction at 2 Gy). The radiosensitizing effect of LBH589 was represented by the dose enhancement factor (DEF), calculated as the ID_50_ for RT treated cells divided by the ID_50_ for combination-treated cells [14]. Two-way ANOVA was used to study the influence of RT dose and LBH589 on the outcome parameters (e.g., survival fraction, cell cycle distribution, γH2AX foci number). A two-sample t test was used to compare the mean percentage of subs G1, G1, S, and G2/M phase cells and the mean γH2AX, BRCA1, BRCA2, RAD51 foci number between specific two treatment conditions. Possible significant differences (*p*<0.05) were evaluated using SPSS v 16.0 software (SPSS, Chicago, IL, USA).

## Results

### The effect of LBH589 alone on cell proliferation using CaP cells and normal prostate epithelial cells

**Figure 1:**
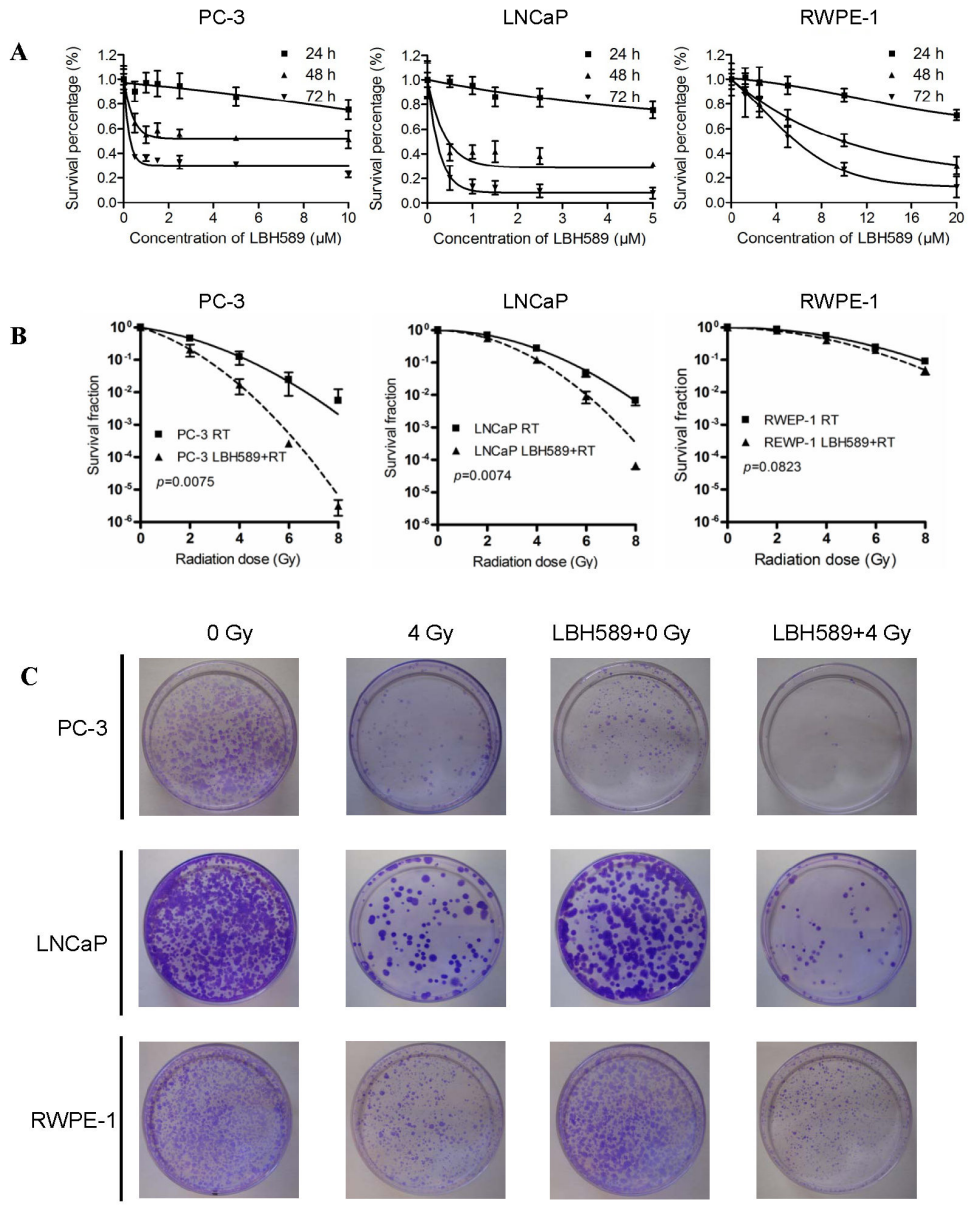
Effect of LBH589 on cell growth and effect of RT or combination treatment (LBH589 and RT) on colony forming in CaP cell lines and normal epithelial prostate cell line. (**A**) Cells were treated with a range of concentrations of LBH589 for 24, 48 and 72 h; (**B**) Cells were treated with RT or combination with RT and LBH589 for analysis of colony-forming efficiency. Survival fractions were significantly reduced in PC-3 and LNCaP CaP cells (*p*<0.01), but not in normal REWP-1 cells (*p*>0.05); (**C**) Typical images are shown for colony growth in RT and combination treatment (LBH589 and RT) in CaP and normal prostate cells. All results were from three independent experiments (N=3). Points, mean; bars, SD.

The time and dose-dependent cell proliferation inhibition effect was found in both CaP cells (PC-3 and LNCaP) and normal prostate epithelial cells (RWPE-1) (Figure **1A**). IC_20_ value at 24 h for PC-3, LNCaP and RWPE-1 was 10 µmol/L, 2.5 µmol/L, and 15 µmol/L, respectively, suggesting that two CaP cell lines are more sensitive to LBH589 than the normal prostate epithelial cell line.

### The effect of combination treatment on colony formation using CaP and normal prostate cells

The radiosensitization effect of LBH589 on PC-3 and LNCaP was indicated by significantly reduced survival fractions (*p*=0.0075 for PC-3, *p*=0.0074 for LNCaP) and DEF in these two cell lines >1 (1.77 for PC-3 and 1.29 for LNCaP) (Figure **1B-C**). Radiosensitivity was not significantly increased in RWPE-1 by LBH589 pretreatment with DEF of 1.18 (*p*=0.0823) (Figure **1B-C**). RT parameters for RT alone and combination treatment in each cell line are summarized in Table **2**. SF2, ID90, ID_50_ and ID10 were all significantly decreased in PC-3 and LNCaP by LBH589 pretreatment (*p*<0.05), but none of these significantly changed for RWPE-1.

**Table 2:** Radiobiological parameters for radiation and LBH589-radiation treatment.

Cell line	Subgroup	α value (Gy^-1^)	β value (Gy^-2^)	α/β value (Gy)	SF2	ID90	ID_50_	ID10	DEF (ratio of ID_50_)
PC-3	Radiation	0.259	0.064	4.047*	0.46*	4.31*	1.84*	0.37*	1.77
	LBH589-radiation	0.542	0.119	4.555	0.21	2.68	1.04	0.19	
LNCaP	Radiation	0.028	0.075	0.373*	0.70*	5.35*	2.86*	1.01*	1.29
	LBH589-radiation	0.053	0.118	0.424	0.56	4.20	2.21	0.75	
RWPE-1	Radiation	1.0E-07	0.038	2.6E-06	0.86	7.78	4.27	1.67	1.18
	LBH589-radiation	0.039	0.042	0.918	0.78	6.92	3.61	1.18	

Abbreviations: SF2 = surviving fraction at 2 Gy; ID90 = radiation dose producing a surviving fraction of 90%; ID50 = radiation dose producing a surviving fraction of 50%; ID10 = radiation dose producing a surviving fraction of 90%; DEF = dose enhancement factor; *p<0.05 vs. corresponding LBH589-radiation treated cells.

### LBH589 alone can induce apoptosis and histone acetylation in CaP cells

Low-dose of LBH589 treatment (IC_20_) for 24 h induced cleavage of full length caspase-3 and acetylation of histone 3 (H3) and histone 4 (H4) in both PC-3 and LNCaP cells (Figure **S1**), suggesting LBH589 may initiate apoptosis pathway related to histone acetylation.

### Effect of combination treatment or RT alone on cell cycle distribution

**Figure 2:**
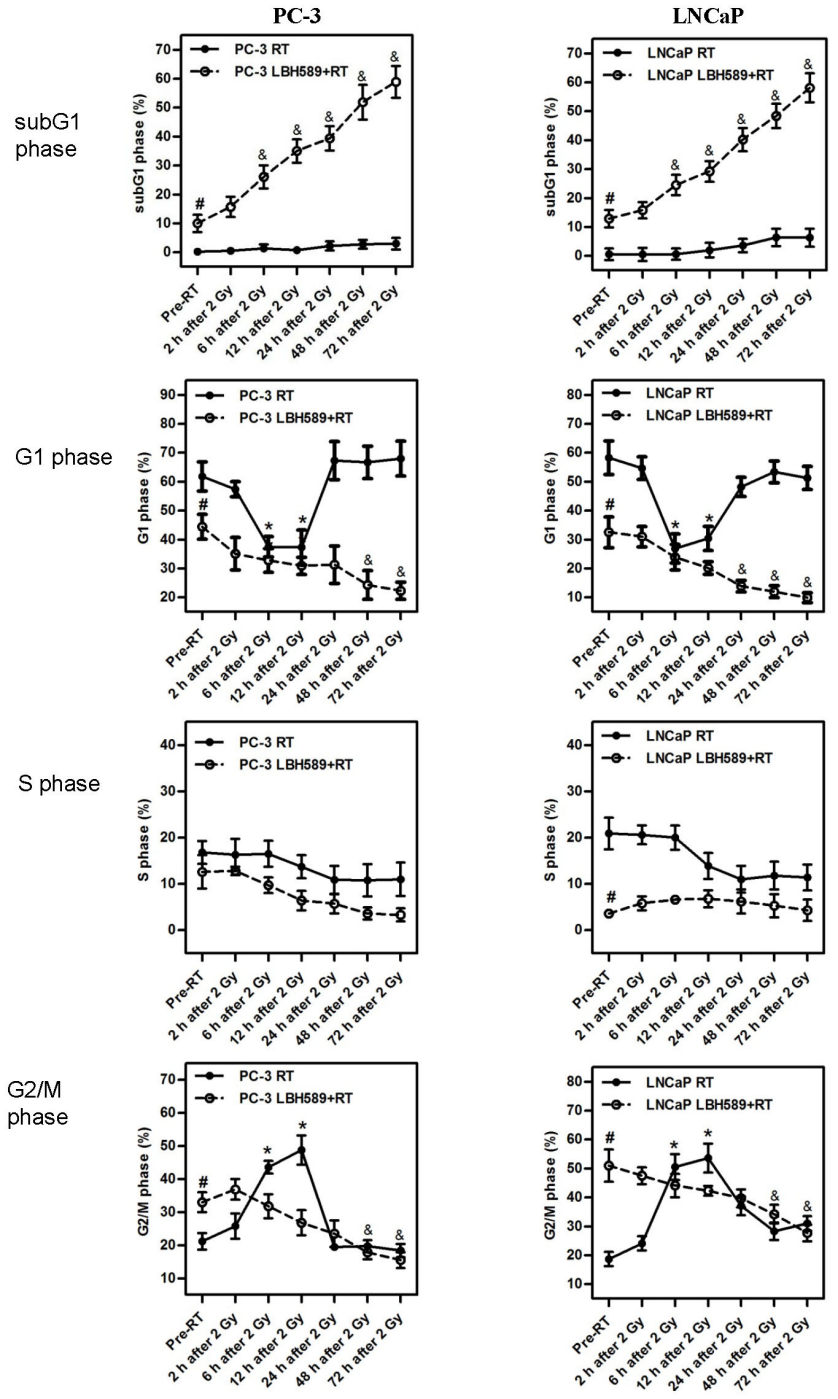
Effects of RT or combination treatment (LBH589 and RT) on cell cycle arrest in PC-3 and LNCaP CaP cell lines. The percentage of subs G1, G1, S, and G2/M populations were analysed using flow cytometry from pre-RT to 72 h post-RT; *p*<0.01(*): significant difference in the percentage of cell cycle phase between the indicated time point and the “Pre-RT” time point in RT groups; *p*<0.01(^#^): significant difference in the percentage of cell cycle phase between the RT group and the combination group at the “Pre-RT” time point; *p*< 0.01(^&^): significant difference in the percentage of cell cycle phase between the indicated time point and the “Pre-RT” time point in combination group. All results were from three independent experiments (N=3). Points, mean; bars, SD.

Percentage of subG1 population increased steadily and significantly in the combination-treated cells compared to RT-treated cells in both CaP cell lines (Figure **2** and Figures **S2**-**S5**), indicating more cell death including apoptosis in combination-treated cells. Upon RT treatment alone, the cells underwent temporary cell cycle delay/G2/M arrest between 2–24 h, and then eventually recovered after 24 h. Accordingly, RT reduced G1 population between 2–24 h, and the G1 resumed after 24 h (Figure **2**). Whereas, in combination treatment (LBH589+RT), the LBH589 pre-treatment on cells initially triggered an augment of G2/M arrest accompanied with the decrease of G1 and S portions. The combination treatment then caused the subsequent and gradual abolishment of all G1, S and G2/M populations of treated cells with a minimum recovery (Figure **2**).

Differences in the G2/M subpopulation between combination-treated and RT-treated groups in each CaP cell line were also observed. Cells that only received 2 Gy RT had significant G2/M arrest 6~12 h after RT in both PC-3 and LNCaP cell lines, and G2/M arrest persisted in the LNCaP cells until 48 h post RT. For combination treated cells, initial LBH589 treatment caused a significant increase of G2/M population percentage prior to RT in both CaP cell lines, and abolished further G2/M arrest after subsequent 2 Gy RT (Figure **2** and Figures **S2**-**S5**).

### Combination treatment can inhibit RT-induced cell cycle checkpoints activation in CaP cells

**Figure 3:**
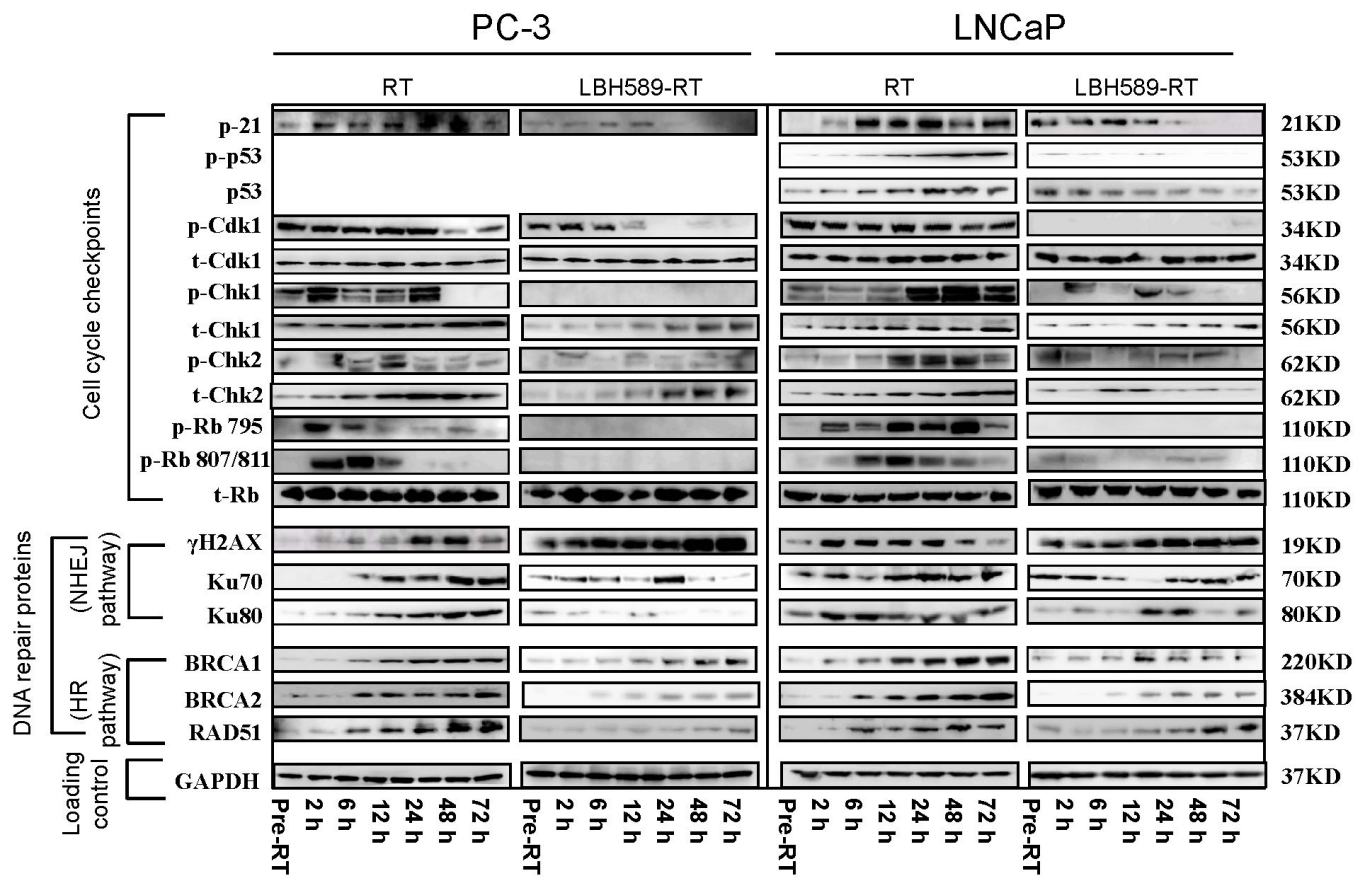
Effects of RT or combination treatment (LBH589 and RT) on cell cycle checkpoints, DNA damage and repair proteins in PC-3 and LNCaP CaP cells from pre-RT to 72 h post-RT. Cell cycle related proteins (p21, p-p53, p53, p-Cdk1, t-Cdk1, p-Chk1, t-Chk1, p-Chk2, t-Chk2, p-Rb795, p-Rb807/811, t-Rb) and DNA damage and repair related proteins (γH2AX, Ku70, Ku80, BRCA1, BRCA2, and RAD51) were determined by western blotting. t: Changes in total protein levels upon DNA damage; p: activation of DNA or cell cycle checkpoint proteins (phosphorylated form). Typical images are shown from three independent experiments (N=3).

The patterns of expression for these checkpoint proteins differed in two CaP cell lines after RT alone or combination treatment with LBH589 and RT are shown in Figure **3**. As p53 is negative in PC-3 cells, the expression of both p21 and p53 increased after 2 Gy RT or combination treatment in LNCaP cells, but they are more persistent in RT treatment alone compared to the combination treatment. The patterns of p21 expression in single or combination treatments in PC-3 cells are very similar to those in LNCaP cells. p21 in either PC-3 or LNCaP cells and p53 in LNCaP cells were not detectable 24 h after combination treatment because LBH589 pretreatment led to gradual decrease of p21 and p53 expression (Figure **3**). Expression of p-CDK1 was observed in RT-treated cells before RT and was persistent until 72 h after 2 Gy RT in both PC-3 and LNCaP cells; in contrast, its expression was down-regulated and even became undetectable in combination-treated groups (Figure **3**). The expression of total-Cdk1 (t-Cdk1) did not change with time, and remained at similar level after single RT and combination treatment (Figure **3**).

The expression levels of total-Chk-1 (t-Chk-1) and total-Chk-2 (t-Chk-2) (two important checkpoint kinases in cell cycle control) upon DNA damage were increasing with post RT time in RT alone group. The addition of LBH589 did not alter the radiation-induced increase in t-Chk-1 and t-Chk-2, but abrogated the increase at all time points (Figure **3**). Combination treatment of LBH589 and RT led to markedly diminished level of activated Chk-1 and Chk-2 (p-Chk-1 and p-Chk-2) after 6 h post RT treatment, as compared to RT treatment alone (Figure **3**). The expression of checkpoint proteins is expected to protect cells from radiation. Our results suggest that LBH589 may affect both the expression and the activity of Chk-1 and Chk-2 thus interfering cell cycle management when combined with RT treatment. p-Rb795 and p-Rb807/811 proteins are important checkpoints responsible for G2/M arrest of cancer cells to RT. Activation of these two proteins was detected in RT-treated cells while no, or very weak expression of these two proteins were detected in combination-treated cells, whereas there was no obvious change detected for the expression of total-Rb (t-Rb) (Figure **3**), which means that LBH589 pretreatment inhibited RT-induced activation of p-Rb795 and p-Rb807/811. Collectively, these results indicate that expression and activation of these cell-cycle related checkpoint proteins are altered in combination-treated groups, compared to RT alone-treated groups.

### Combination treatment can induce persistent DNA double strand break (DSB) in CaP cells

**Figure 4:**
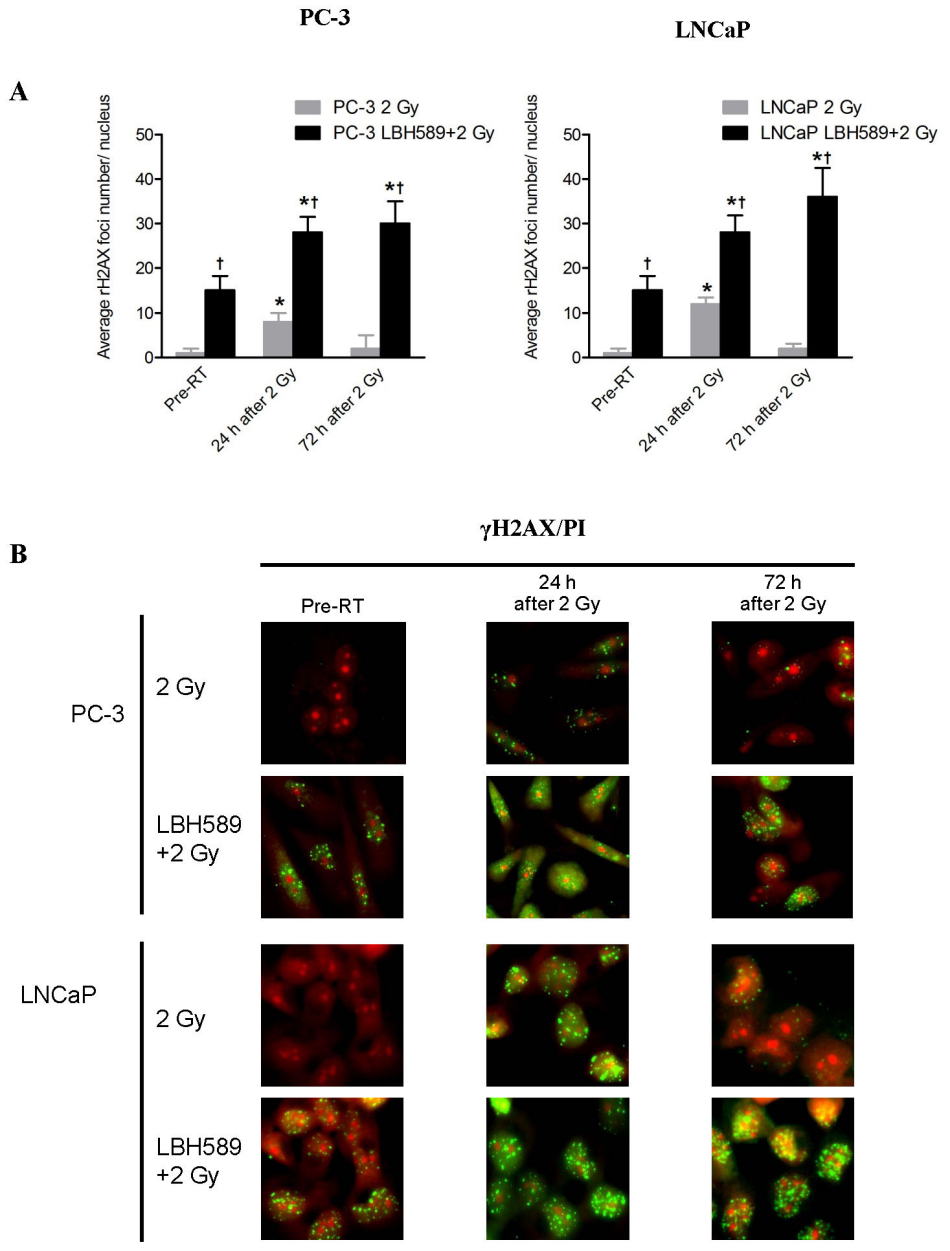
Quantitative analysis of DNA double strand break (DSB) marker γH2AX foci at pre-RT, 24 h and 72 h after 2 Gy RT or combination treatment in PC-3 and LNCaP cells. (**A**) After 2 Gy RT, average γH2AX foci number increased significantly at 24 h in both PC-3 and LNCaP cells and decreased to a lower level at 72 h while average γH2AX foci number in combination-treated (LBH589+2 Gy RT) cells was significantly higher than that in RT-treated cells at any time points and continued to increase until 72 h. (**B**) Representative images of γH2AX staining after 2 Gy RT or combination treatment (LBH589+2 Gy RT) in PC-3 and LNCaP cells. *p*<0.05(†) indicates significant difference between RT-treated cells and combination-treated cells at the same time point. *p*<0.05(*) indicates significant difference between cells at specific time points and cells receiving the same treatment at the “Pre-RT” time point. Points, mean; bars, SD. N=50. Typical images are shown from three independent experiments (N=3). Magnification × 60 in all images.

DNA DSB was examined using γH2AX as a marker by western blotting and fluorescence staining. In 2 Gy RT-treated CaP cells, the expression of γH2AX started to increase from 10–15 min after RT, peaking at 48 h and 2 h, for PC3 and LNCaP respectively, as shown by western blotting (Figure **3**). The γH2AX results from western blotting were further confirmed by immunofluorescence staining (Figure **4**). In contrast, for the cells treated with combination therapy, γH2AX was moderately expressed at the pre-RT time point and was up-regulated on the time scale in both PC-3 and LNCaP cells until 72 h (Figure **3**). Number of γH2AX foci in nuclei was quantified as shown in Figure **4**. Positive γH2AX foci were still detectable 72 h after 2 Gy RT in PC-3 and LNCaP cells by combination treatment. These results indicate that DNA DSB persists after combination treatment compared to RT alone.

### Combination treatment can decrease activation of non-homologous end joining (NHEJ) repair pathway in CaP cells

Since persistent DNA DSB were seen in combination-treated cells, two NHEJ pathway proteins (Ku70 and Ku80) which are responsible for repair of RT-induced DSB were further examined by western blotting in RT- and combination-treated cells. Expression patterns of Ku70 and Ku80 were different for PC-3 and LNCaP cells (Figure **3**). In PC-3 cells, positive expression of Ku70 and Ku80 was found 6 h after RT and increased gradually until 72 h after RT whereas expression of Ku70 and Ku80 was undetectable after 48 h in combination treatment (Figure **3**). In LNCaP cells, positive expression of Ku70 and Ku80 was found at all time points for RT alone and combination treatment with LBH589 and RT, but expression levels of Ku70 and Ku80 were much higher after RT alone compared to combination treatment at all time points (Figure **3**). These results indicate NHEJ pathway was involved in RT in CaP cells and that pre-treatment with LBH589 can decrease its activation compared to RT alone.

### Combination treatment can decrease activation of homologous recombination (HR) repair pathway in CaP cells

**Figure 5:**
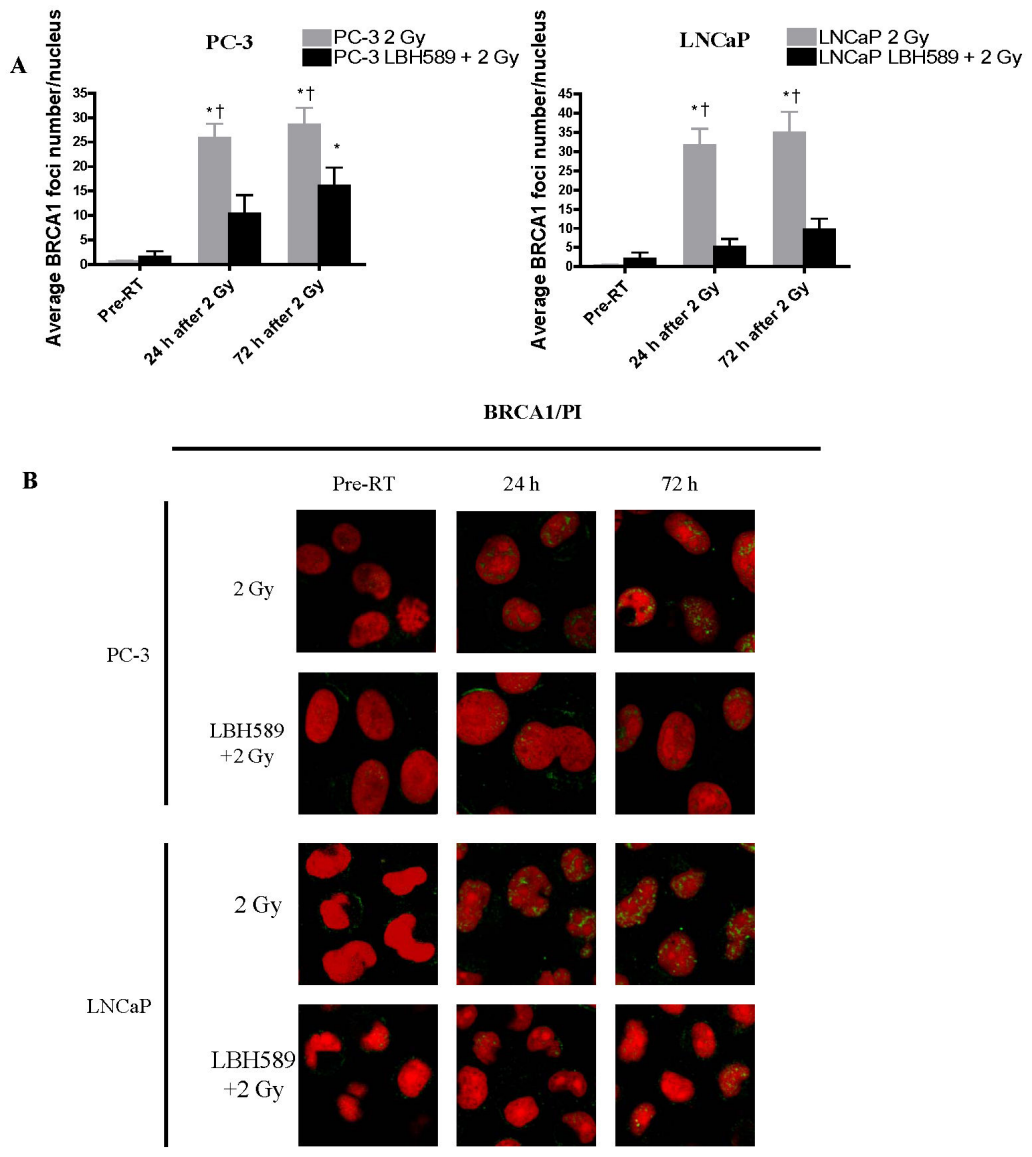
Quantitative analysis of DSB repair marker in the HR pathway for BRCA1 foci at pre-RT, 24 h and 72 h after 2 Gy RT or combination treatment in PC-3 and LNCaP cells. (**A**) After 2 Gy RT, average BRCA1 foci number increased significantly at 24 h in both PC-3 and LNCaP cells and maintained increased at 72 h, while average BRCA1 foci number in combination-treated (LBH589+2 Gy RT) cells was also increased at 24 and 72 h, but was significantly lower than that in RT-treated cells. (**B**) Representative images of BRCA1 staining after 2 Gy RT or combination treatment (LBH589+2 Gy RT) in PC-3 and LNCaP cells. *p*<0.05(†) indicates significant difference between RT-treated cells and combination-treated cells at the same time point. *p*<0.05(*) indicates significant difference between cells at specific time points and cells receiving the same treatment at the “Pre-RT” time point. Points, mean; bars, SD. N=50. Typical images are shown from three independent experiments (N=3). Magnification × 60 in all images.

**Figure 6:**
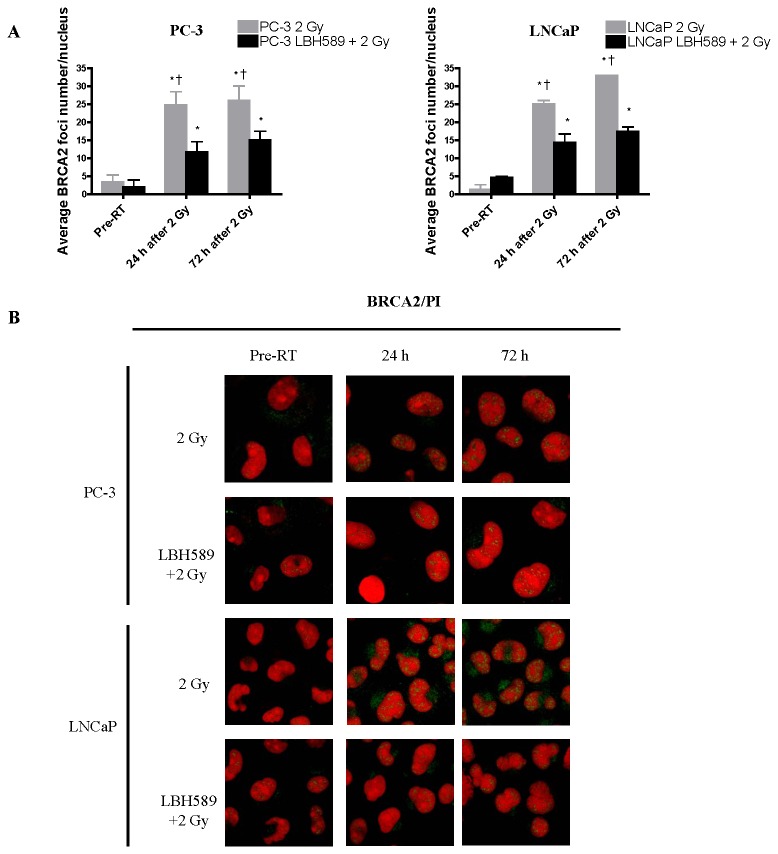
Quantitative analysis of DSB repair marker in the HR pathway for BRCA2 foci at pre-RT, 24 h and 72 h after 2 Gy RT or combination treatment in PC-3 and LNCaP cells. (**A**) After 2 Gy RT, average BRCA2 foci number increased significantly at 24 h in both PC-3 and LNCaP cells and maintained increased at 72 h, while average BRCA1 foci number in combination-treated (LBH589+2 Gy RT) cells was also increased at 24 and 72 h, but was significantly lower than that in RT-treated cells. (**B**) Representative images of BRCA2 staining after 2 Gy RT or combination treatment (LBH589+2 Gy RT) in PC-3 and LNCaP cells. *p*<0.05(†) indicates significant difference between RT-treated cells and combination-treated cells at the same time point. *p*<0.05(*) indicates significant difference between cells at specific time points and cells receiving the same treatment at the “Pre-RT” time point. Points, mean; bars, SD. N=50. Typical images are shown from three independent experiments (N=3). Magnification × 60 in all images.

**Figure 7:**
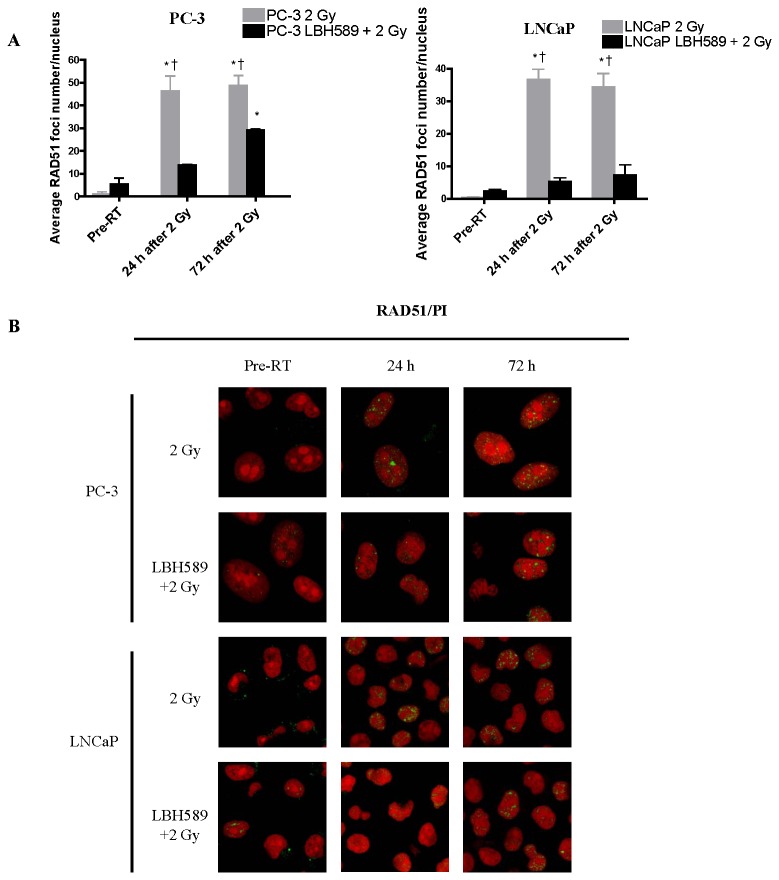
Quantitative analysis of DSB repair marker in the HR pathway for RAD51 foci at pre-RT, 24 h and 72 h after 2 Gy RT or combination treatment in PC-3 and LNCaP cells. (**A**) After 2 Gy RT, average RAD51 foci number increased significantly at 24 and 72 h in both PC-3 and LNCaP cells, while average BRCA1 foci number in combination-treated (LBH589+2 Gy RT) cells was also increased at 24 and 72 h, but was significantly lower than that in RT-treated cells. (**B**) Representative images of RAD51 staining after 2 Gy RT or combination treatment (LBH589+2 Gy RT) in PC-3 and LNCaP cells. *p*<0.05(†) indicates significant difference between RT-treated cells and combination-treated cells at the same time point. *p*<0.05(*) indicates significant difference between cells at specific time points and cells receiving the same treatment at the “Pre-RT” time point. Points, mean; bars, SD. N=50. Typical images are shown from three independent experiments (N=3). Magnification × 60 in all images.

BRCA-1, BRCA-2 and Rad-51 are the DSB repair proteins in the HR pathway. The expression of these proteins was increasingly induced from 2 h until 72 h after single RT by western blotting (Figure **3**). Compared to the RT alone group, the expression of BRCA-1 and BRCA-2 proteins in the combination treatment groups followed the same trend but was abrogated after 6 h post RT in both cell lines. Especially, the expression of RAD51 protein was maintained in a lower level until 72 h in PC-3 cells and was markedly reduced until 24 h in LNCaP cells in the combination groups (Figure **3**). The BRCA-1, BRCA-2 and Rad-51 results from western blotting were further confirmed by immunofluorescence staining (Figures **5**-**7**). Number of the foci from three protein repair markers in nuclei was quantified as shown in Figures **5**-**7**. The results from the western blot were consistent with the immunofluorescent staining results. Our findings suggest that in addition to affecting NHEJ repair pathway, LBH589 may also sensitize RT via interfering HR pathway thus weakening DSB repair ability of the CaP cells.

## Discussion

In this study, we demonstrated that LBH589 inhibited the growth of PC-3, LNCaP CaP cells and RWPE-1 normal prostate epithelial cells in a dose and time-dependent manner, which is similar to previously reported toxicities of other hydroxamates [15]. The normal prostatic epithelial cell line RWPE-1 was the most resistant to LBH589 while LNCaP was the most sensitive among the three cell lines, suggesting CaP cells are relatively sensitive to LBH589.

It has been recognized that α/β value is substantially lower in CaP than most other cancers. The α/β values of PC-3 and LNCaP cells in the current study are in line with previous reports, which is between 0.5 and 4 Gy [16]. When cells received combination treatment (LBH589 and RT), the α/β values of PC-3 and LNCaP cells was increased to 4.555 and 0.424 Gy, respectively. DEF was 1.77 for PC-3 and 1.29 for LNCaP. The α/β value of RWPE-1 normal prostate cell line was 0.243 and 0.257 Gy after RT alone or combined treatment, which is correspondingly lower than the two CaP cell lines. DEF was 1.18 which is less than that in PC-3 and LNCaP cell lines. These results warrant further *in vivo* study and clinical trials.

In the current study, our results demonstrated that even low dose of LBH589 (IC_20_) for 24 h treatment could trigger apoptosis in CaP cells (Figure **S1**) and the percentage of the subG1 population cells in combination treatment of LBH589 and RT gradually increased while it was consistently quite low in the RT treated cells, meaning that combination treatment can induce more cell death.

Cell cycle analysis further confirmed that more PC-3 and LNCaP cells were blocked in G2/M phase after 24 h LBH589 treatment and the percentage of cells in G1 phase decreased significantly. Moreover, treatment with LBH589 for 24 h reduced the S-phase content of LNCaP cells to a lower level. In all the cell cycle phases, G2/M phase cells are the most sensitive to radiation and S phase cells are the most resistant [17]. Our results suggest that cell cycle arrest at G2/M and decrease of S phase percentage might be responsible for the radiosensitization effect of LBH589. The p53-p21 axis plays a very important role in the regulation of cell cycle in CaP RT [18,19]. The check point proteins Chk1/2-mediated p53 phosphoralation can result in the activation of p21 transcription, thereby inhibits Cdk activity and leads to cell cycle arrest at G1 phase [20]. It was reported that up-regulation of p21 and its subsequent binding to the CDK1-cyclin complexes [21] inhibit CDK1 phosphorylation and lead to a G2/M cell cycle arrest effect [22]. Here, we found that in the later time points (24 h, 48 h, 72 h), both p53 (PC-3 cell line is p53 negative) and p21 proteins were lower in the combination treatment compared to those treated with RT alone, which is in accordance with the G1 defect in cell cycle following the combination treatment in the later time points. The initial increase of p21, p53 and reduction of p-CDK1 after LBH589 treatment especially in LNCaP cells indicate a potential mechanism for G2/M arrest caused by LBH589. The deficiency of p53 in PC-3 cells suggests that p21 may be regulated by alternative mechanisms.

After radiation, G2/M arrest is a protective reaction which enables cells to repair DNA damage before entering mitosis. G2/M arrest caused by 2 Gy RT wasn’t detected in the combination group. Correspondingly two cell cycle checkpoints, p-Rb795 and p-Rb807/811, were activated after 2 Gy RT, but combination treatment significantly reduced all their activation in both PC-3 and LNCaP cells, indicating LBH589 treatment at IC_20_ concentrations were effective to perturb CaP cells’ regulation of cell cycle after RT. These results may also explain part of the radiosensitization effect of LBH589.

In our study, expression of γH2AX was enhanced by combination treatment with LBH589 and RT in both PC-3 and LNCaP cells, including LBH589-induced endogenous foci before radiation as well as promoting the radiation-induced foci. NHEJ and HR pathways are the most important two signaling pathways responsible for repairing DNA DSBs. We found for the first time that key proteins including Ku70 and Ku80 are activated in PC-3 and LNCaP cells after RT and that both Ku70 and Ku80 proteins were less activated in CaP cells by LBH589 pretreatment, which implies that the NHEJ repair pathway plays an important role in the regulation of CaP radiosensitivity after exposure to RT.

HR pathway repairs DSB using a homologous chromatid or chromosome [23]. It was reported that only ~10% of DSB were repaired by the HR pathway in mammalian cells [24]. In the current study, we have shown that the BRCA1, BRCA2 and Rad51 proteins which are the main partakers in the HR pathway, were presented with the delayed increase in the combination treatment group, when compared to RT alone group. Therefore, these HR repair pathway proteins are involved in the radiosensitization effect induced by LBH589. Given that LBH589 is a pan-HDACi, it may interfere with activation of all these proteins by increasing acetylation. This could be the possible reason why the DNA DSBs repair was significantly impaired in the combination-treated CaP cells. Our results are consistent with the observation by other group that most of genes involved in cell cycle control, DNA replication and DNA damage repair were downregulated after treatment with HDACi [25]. Our findings indicate that both NHEJ and HR repair pathways are involved in DNA repair defective in the combination treatment.

The loss of histone lysine acetylation has been observed to be related with carcinogenesis. Over the past decade, HDAC, which is responsible for removing the acety group from histones, have evolved as one of the major cancer targets for epigenic based therapies [26]. Combining epigenic therapy with HDACi or traditional regimens such as chemotherapy or RT is a new developing research area in reducing toxicity in chemotherapy and radiotherapy [27–30]. Like other HDACis, the LBH589 (HDACi)-mediated radiosensitization might result from several mechanisms: 1) the expression of check point and DSB repair proteins were inhibited by HDACi [25]; 2) HDACi mediated deacetylation may cause disruption of protein–protein interaction during cell cycle control and DSB repair [31]; 3) the HDACi treatment resolves the compact structure of the chromatin, which may lead to the susceptibility of the cells to radiation damage [32] and the activation of specific gene transcription [33,34]. All above reasons may cause the defects in DNA damage repair and checkpoint proteins which would allow the cells to proceed through the cell cycle with damaged DNA, resulting in more apoptosis. This phenomenon has been shown for cell death upon chemo- and radiotherapy treatment in several cancers [35]. Our study indicates that LBH589, even in a low dose, is a potent radiosensitizer in CaP cells *in vitro*, and cell cycle check point and DSB repair proteins (p21, p53, Cdk1, Chk1, Chk2, Rb, γH2AX, Ku70, Ku80, BRCA-1, BRCA-2, and RAD51) are activated in response to RT alone so that the cells may gain sufficient time for DNA repair. After CaP cells received the combination therapy (LBH589+RT), the activation of these proteins were inhibited, leading to DNA repair defect and the increase of radiosensitivity of CaP cells.

In summary, our results demonstrated for the first time that LBH589 at low concentration (IC_20_) sensitizes CaP cells to RT. The putative mechanisms of the radiosensitization effect in CaP cells include induction of apoptosis; redistribution to a more radiosensitive cell cycle phase and abolishment of RT-induced cell cycle arrest; induction of more DNA damage and inhibition of repair of RT-induced DNA DSBs through diminishing NHEJ and HR pathways. Combination of LBH589 and RT may provide a new treatment strategy to improve anti-cancer efficacy while reducing the toxicity of extreme high dose RT.

## Supporting Information

Figure S1 **Effects of LBH589 on expression of apoptotic proteins and acetylated histones in CaP cells.** After LBH589 treatment at IC_20_ concentrations for 24 h, expression of Caspase-3 (Pro), Caspase-3 (Active)], acetylated H3 and acetylated H4 was determined by western blotting in PC-3 and LNCaP cells. The typical images are shown from three independent experiments (N=3).
(TIF)

Figure S2 **Representative images of cell cycle histograms of PC-3 cells after 2 Gy RT from 0- to 72 h post-RT.**
(TIF)

Figure S3 **Representative images of cell cycle histograms of PC-3 cells after combination treatment of LBH589 and 2 Gy RT from 0 to 72 h post-RT.**
(TIF)

Figure S4 **Representative images of cell cycle histograms of LNCaP cells after 2 Gy RT from 0 to 72 h post-RT.**
(TIF)

Figure S5 **Representative images of cell cycle histograms of LNCaP cells after combination treatment of LBH589 and 2 Gy RT from 0 to 72 h post-RT.**
(TIF)
