# Supplementary figures and images for: Combination Therapy with the Histone Deacetylase Inhibitor LBH589 and Radiation Is an Effective Regimen for Prostate Cancer Cells
Source: PLoS One. 2013 Aug 26;8(8):e74253. doi: 10.1371/journal.pone.0074253 (PMC3753304; doi:10.1371/journal.pone.0074253)

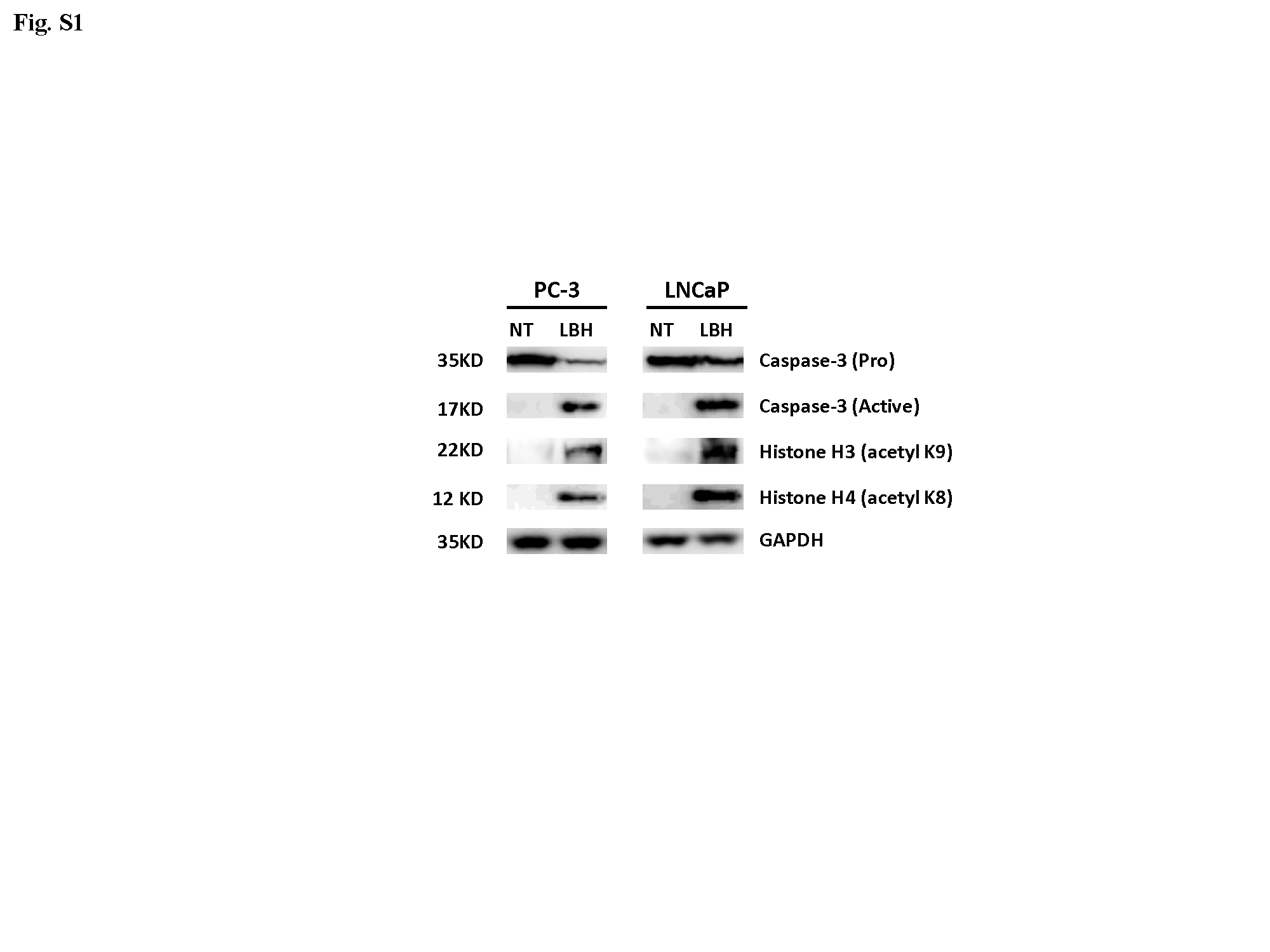

Supplement: Figure S1 — Effects of LBH589 on expression of apoptotic proteins and acetylated histones in CaP cells. After LBH589 treatment at IC20 concentrations for 24 h, expression of Caspase-3 (Pro), Caspase-3 (Active)], acetylated H3 and acetylated H4 was determined by western blotting in PC-3 and LNCaP cells. The typical images are shown from three independent experiments (N=3). (TIF) [file pone.0074253.s001.tif]

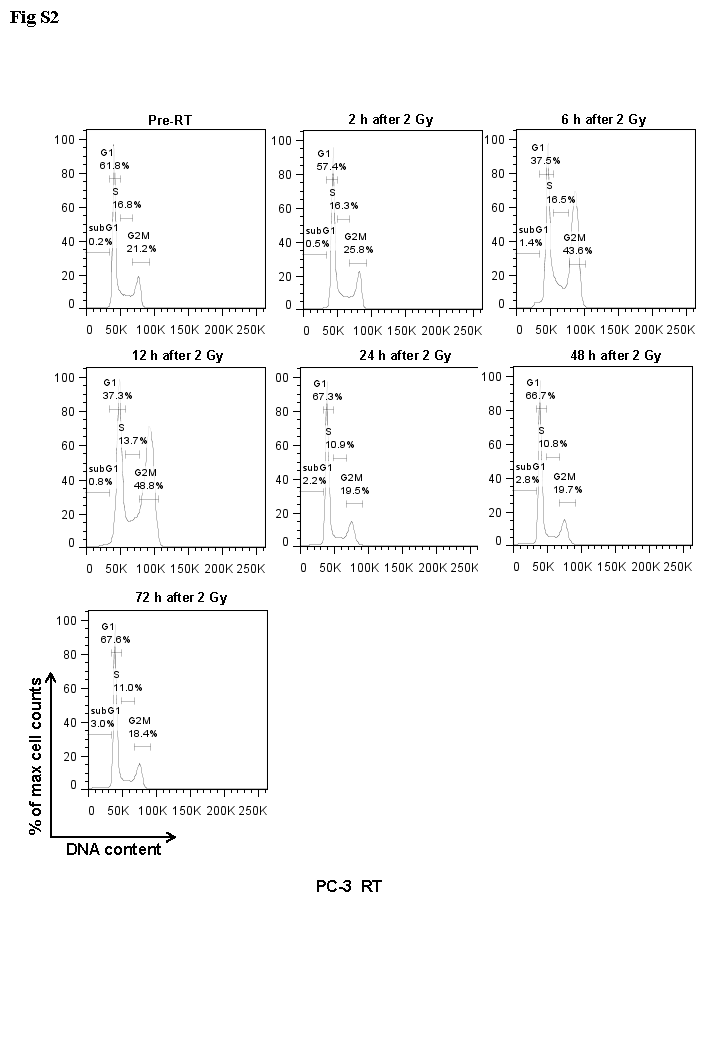

Supplement: Figure S2 — Representative images of cell cycle histograms of PC-3 cells after 2 Gy RT from 0- to 72 h post-RT. (TIF) [file pone.0074253.s002.tif]

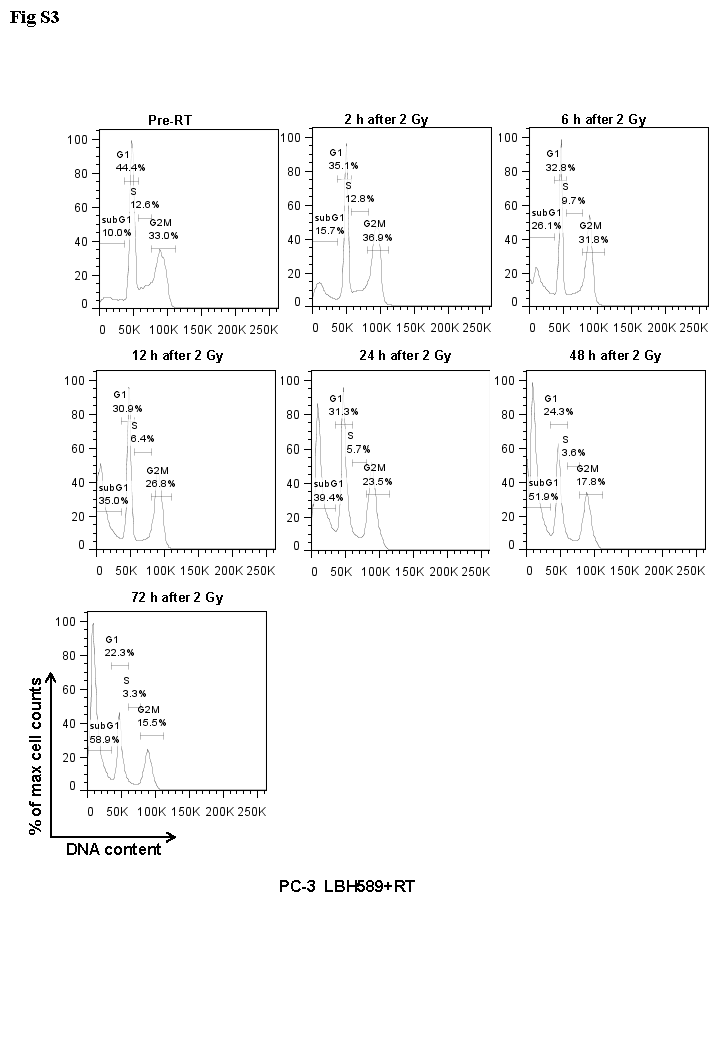

Supplement: Figure S3 — Representative images of cell cycle histograms of PC-3 cells after combination treatment of LBH589 and 2 Gy RT from 0 to 72 h post-RT. (TIF) [file pone.0074253.s003.tif]

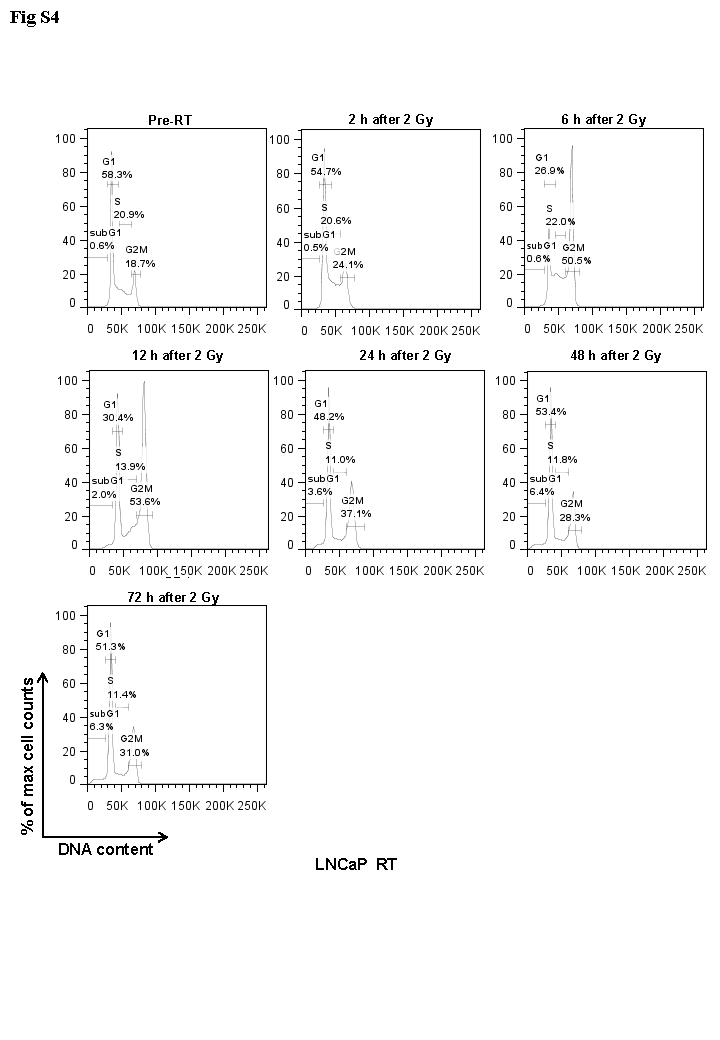

Supplement: Figure S4 — Representative images of cell cycle histograms of LNCaP cells after 2 Gy RT from 0 to 72 h post-RT. (TIF) [file pone.0074253.s004.tif]

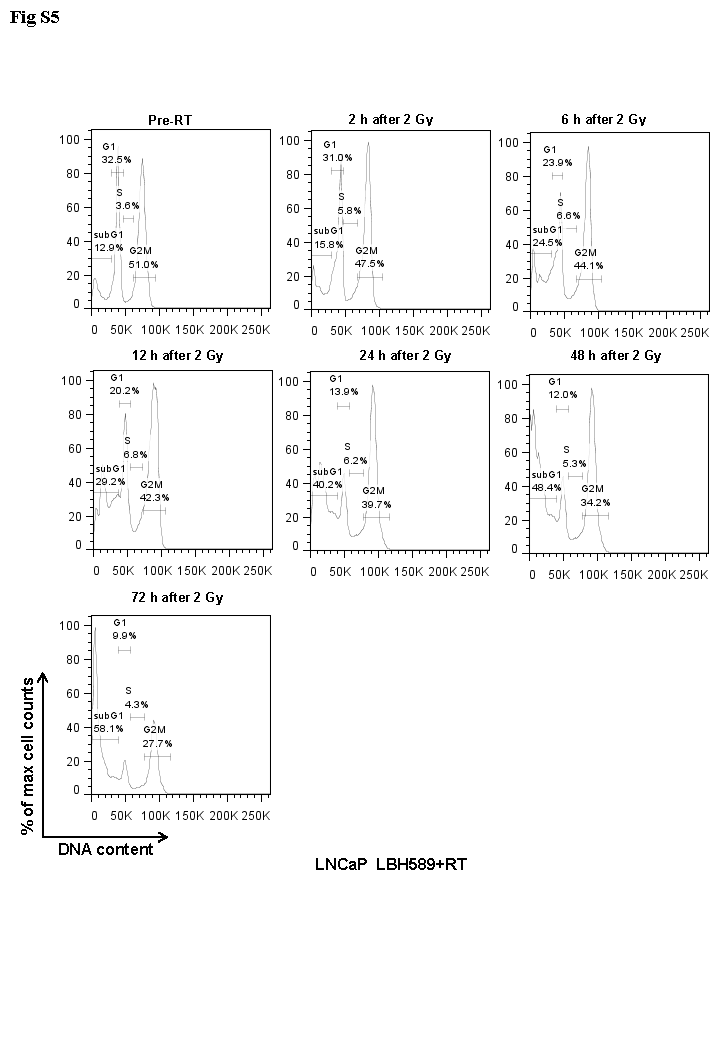

Supplement: Figure S5 — Representative images of cell cycle histograms of LNCaP cells after combination treatment of LBH589 and 2 Gy RT from 0 to 72 h post-RT. (TIF) [file pone.0074253.s005.tif]
